# Are We Ready to Measure Skin Permeation of Modern Antiaging GHK–Cu Tripeptide Encapsulated in Liposomes?

**DOI:** 10.3390/molecules30010136

**Published:** 2025-01-01

**Authors:** Karolina Ogórek, Kinga Nowak, Emilia Wadych, Lena Ruzik, Andrei R. Timerbaev, Magdalena Matczuk

**Affiliations:** 1 Faculty of Chemistry, Warsaw University of Technology, Noakowskiego St. 3, 00-664 Warsaw, Polandkinga.nowak3.stud@pw.edu.pl (K.N.); emilia.wadych.stud@pw.edu.pl (E.W.); lena.ruzik@pw.edu.pl (L.R.); 2 Institute of Inorganic Chemistry, University of Vienna; Währinger Str. 42, 1090 Vienna, Austria

**Keywords:** GHK–Cu, skin permeation, liposomes

## Abstract

Cosmetically active compounds (CACs), both of lipophilic and hydrophilic origin, have difficulty reaching the deeper layers of the skin, and this shortcoming significantly reduces their efficacy. One such CAC that occurs naturally in the human body and displays many beneficial properties (via reducing fine lines and wrinkles, tightening skin, improving its elasticity, etc.) is the glycyl-L-histidyl-L-lysine tripeptide complex of copper (GHK–Cu). GHK–Cu is a fairly hydrophilic compound with limited permeation through the lipophilic stratum corneum. On the other hand, liposomes capable of encapsulating GHK–Cu may improve its permeation potential. The present review discusses various issues related to obtaining insight into the permeation of CACs through the skin. Methods for studying the transport of CACs encapsulated by liposomes and free GHK–Cu across the skin barrier are summarized. An analysis of the literature data reveals that the transport of liposomes containing GHK–Cu received little attention. This research gap gives an impetus to the methodological developments for assessing the effect of liposomes on GHK–Cu transportation and trafficking.

## 1. Introduction

Skin, the human body’s largest structure, consists of three main layers: the epidermis, dermis, and subcutaneous tissue [1]. In the frames of this work, the most important is the outer layer of the skin, the epidermis defined as a multilayered squamous epithelium. Its outermost part is the stratum corneum, which acts as the primary barrier protecting the body from harmful environmental factors. At the same time, it hinders the permeation of active ingredients that may benefit the skin condition and the health of the whole body [2]. Therefore, a key element in improving cosmetic formulations is research into the skin permeation of compounds with therapeutic effects. Various methods are known for determining skin permeation, but the most advantageous are in vitro approaches as an alternative to more demanding human and animal testing.

**Figure 1:**
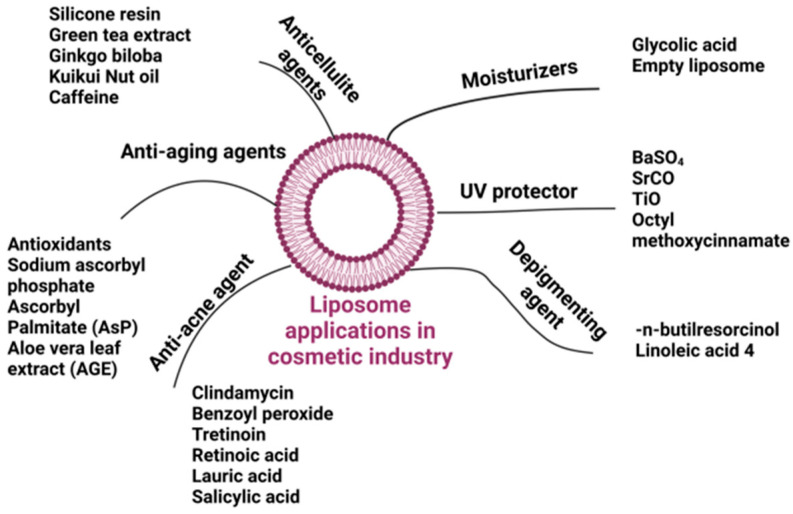
Application of liposomes in the cosmetic industry [5].

The cosmetics industry faces many challenges, especially when boosting the permeation of cosmetically active compounds (CACs) through the skin is an issue. Their physicochemical properties, such as the size and solubility in fats or water, significantly influence the permeation ability. Nanotechnology presents new opportunities to enhance skin permeation, although further research is required to ensure safety and efficiency of the employed nanomaterials. In this context, using liposomes as carriers may not only help overcome the skin barrier and but also improve the bioavailability of CACs. Liposomes are nanometric self-assembling vesicles composed of phospholipids. In an aqueous solution, the phospholipids (consisting of a hydrophilic head and a hydrophobic tail) concentrate the hydrophilic heads toward the aqueous medium, thus forming double layers surrounding the aqueous core [3]. Due to their biocompatibility, non-toxicity, and morphological similarity to living cell membranes, liposomes have been widely used in products intended for use on the skin. Their structure allows for the effective delivery of active ingredients into the deeper layers of the epidermis while protecting them from degradation, increasing their stability and efficacy. The introduction of the first liposomal antiaging cream under the Christian Dior brand in 1986 triggered intensive research of new liposomal formulations [4]. Today, the cosmetic industry successfully uses liposomes in various products, including moisturizing, wrinkle-reducing, antiaging, UV protecting, depigmenting, and anti-acne formulations (Figure 1) [5].

It has been confirmed that liposomes can serve as carriers for various active compounds, including vitamins, antioxidants, and organic acids. Specifically, the skin penetration of vitamin C [6,7,8], vitamin D3 [9], coenzyme Q10 [10], and caffeine [11] encapsulated in various liposomes has been studied. While some CACs have been successfully encapsulated in liposomes, the extent of their skin permeation and release from these carriers has not been examined. One such compound is the modern antiaging ingredient, GHK–Cu, which exhibits multiple beneficial effects but poorly penetrates the lipophilic stratum corneum. Although GHK–Cu tends to be effectively encapsulated in liposomes [12,13,14,15,16,17], its skin permeation has only been scrutinized using a free form. For this reason, it seems necessary to critically assess the contemporary tools capable of evaluating the penetration of liposomal GHK–Cu through human skin, and this is the major goal of this review.

## 2. Transdermal Delivery of CACs

**Figure 2:**
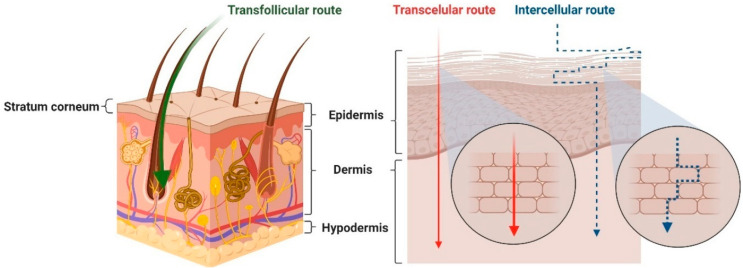
Skin penetration routes [19].

The CACs exert an impact on the physiology and functioning of the skin and the entire body. However, before reaching the targets, they have trafficked a long way. The CAC penetration through the skin takes place in three steps: adsorption, by which the CAC reaches the stratum corneum; penetration or entering into the dermis; and resorption, which leads to the CAC transport into the blood vessels [18]. CACs may enter the deeper layers of the skin by various routes (Figure 2).

These include the following:

- Intercellular transport—A transport pathway that is continuous and winding through the lipid matrix between corneocytes. The intercellular path is considered the main transport route for small, uncharged, and lipophilic molecules [20].
- Transcellular transport—Involves the passage of CACs through the relatively hydrophilic environment of the corneocytes and then through the highly lipophilic intercellular lipid matrix. This pathway provides the shortest route and is preferred mainly for the transport of hydrophilic molecules [21].
- Transfollicular transport (through hair follicles and sebaceous gland mouths)—A highly permeable pathway following a transepithelial route. It is used to transport polar or ionizable compounds and large macromolecules, which have difficulty passing through the epidermal cells due to their size and different partitioning properties. However, given that appendages occupy only 0.1% of the total skin surface, they account for a deficient proportion of the total transport of compounds through the skin [22,23].

**Figure 3:**
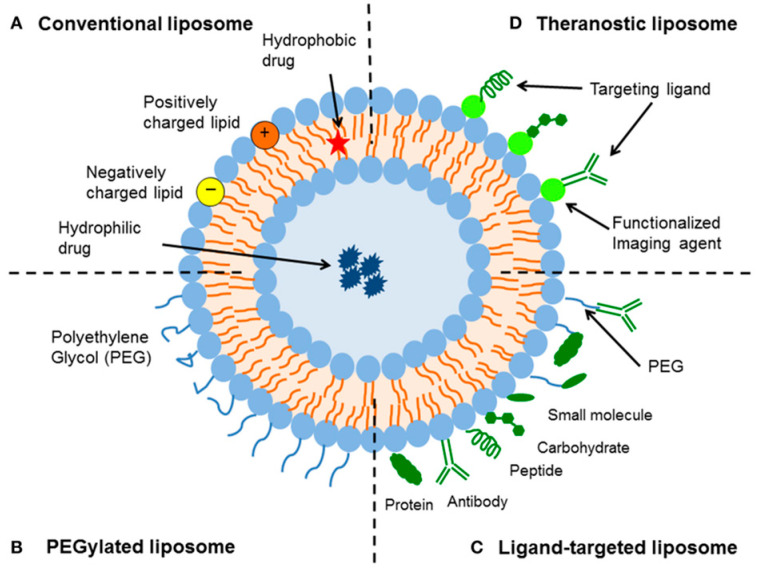
Types of liposomal delivery systems [25].

The rate at which the CAC traffics to its destination depends mainly on the stratum corneum, the main barrier during absorption through the skin, as well as on the properties of the CAC itself. It is possible to increase the penetration by chemical modification of CACs, modification of the epidermis, addition of permeation promoters, or using different carriers [24]. Liposomes have proven as first-in-class carriers for CACs applied to the skin (Figure 3). Delivery of the CAC via conventional liposomes is affected by several factors, including liposome size, surface charge, lamellarity, lipid composition, and the thermodynamic state of the lipid bilayer [25,26]. Liposomal carriers tend to transport and accumulate CACs in the upper layers of the skin, and research toward improving their penetration ability has confirmed the critical influence of various structural modifications. In response, new classes of phospholipid carriers, mostly transferosomes and ethosomes, have received increased attention. Transferosomes due to their deformability and flexibility (owing to incorporating edge activators) can transport compounds more efficiently [27,28]. In the case of ethosomes, the high ethanol content improves the fluidity of the phospholipid bilayer [29]. In addition to these modified vesicles, other carriers, such as hyalurosomes (sodium hyaluronate immobilized vesicles) [30,31], glycerosomes (consisting of phospholipids, water, and a high content of glycerol) [32,33], and a penetration enhancer containing vesicles, have been pursued [33,34,35,36].

The mechanism of action of liposomes is still a matter of debate [23,37]. Some authors insist that liposomes can act as carriers, penetrating intact through the skin. Others propose that liposomes perform the function of penetration enhancers because when applied onto the skin, they break down the cell membranes and diffuse as a lipid mixture. According to other findings, liposomes modulate the penetration of both encapsulated and unencapsulated compounds [37,38].

## 3. The State of Skin Permeation Measurements

**Figure 4:**
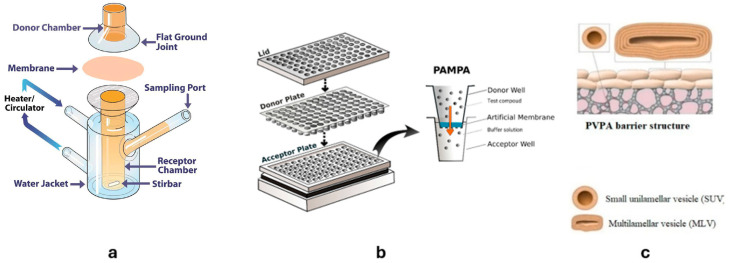
Schemes of (**a**) a Franz diffusion cell [41]; (**b**) Skin-PAMPA [42]; and (**c**) PVPA barrier [43].

The industry is constantly trying to improve the effectiveness of cosmetic products while maintaining their safety. Ongoing developments, including using various carriers, give rise to an acute need to understand the transportation mechanisms through the skin. The product’s efficacy and the degree of therapeutic response depend mainly on how efficiently the CAC is released from the carrier and then penetrate the epidermis and deeper skin layers [39]. Although modeling the transportation mechanism is challenging, the knowledge acquired rewards us by endorsing the development of new cosmetics. There are several methods for testing the permeation and release of CACs, which can be divided into in vitro, ex vivo, and in vivo methods (i.e., testing directly on whole human or animal). The most commonly used approach is in vitro or ex vivo tests using diffusion cells, or more recently, the parallel artificial membrane permeability assay (Skin-PAMPA) and phospholipid vesicle-based permeation assay (PVPA) (Figure 4) [40], allowing for the quantification of the permeation of CACs through the skin.

As shown in the figure, Franz diffusion cells (FDSs) typically have a vertical arrangement, with each cell comprising a donor and an acceptor compartment separated by a membrane mimicking human skin. The choice of the appropriate membrane significantly impacts the outcome of the tests conducted. FDSs are designed to probe formulations such as creams, emulsions, or gels [44]. The preparation is applied in the donor compartment directly on the membrane, and the acceptor compartment is filled with a suitable solution. In the case of testing CACs, it is usually a pH 7.4 buffer, mimicking the physiological environment. The solution in the acceptor compartment is constantly stirred [6,10]. Less commonly used are horizontal cells, called Side-Bi-Side cells, aimed at testing the CACs in the form of solutions [45]. On the contrary, the FDSs exhibit better suitability due to operating under typical skin permeation conditions since the tested formulation is placed directly on the membrane, which is virtually the same as the skin.

Another valuable approach is based on using the diffusion flow cell, also known as the Bronaugh cell. The main difference from the FDS is that the buffer flows continuously through the acceptor compartment. Diffusion flow cells are often utilized to explore the permeation of CACs with high absorption and low solubility in the acceptor phase. Before the test, a minimum phase flow rate should be determined experimentally [46]. The advantage of the Bronaugh cell is the continuous perfusion of the underside of the skin (dermis) with fresh receptor fluid to maintain the absorption conditions. Conversely, continuous fluid flow can change the properties of the skin and lead to overestimated values of permeability.

The commercially available Skin-PAMPA test, conducted using 96-well plates (see Figure 4b), is a novel but not yet frequently used tool for measuring skin permeability. The wells in the lower plate are filled with a suitable acceptor solution, while the upper plate has a ready-made membrane in each well, which consists of ceramides, free fatty acids, and sterols. The PAMPA offers a simple and rapid way to assess the permeability of human skin and the affinity of CACs for the stratum corneum [47]. This method has been used to test the permeability of preparations containing ibuprofen [48], niacinamide [49], diclofenac sodium [50], and retinol [51], among others. Alternatively, a permeation test based on phospholipid vesicles can be used with a membrane, imitating the skin, prepared by tightly placing layers of liposomes on the filter [52].

Relevant permeability information can also be obtained using microscopic techniques. These include two-photon microscopy [53], confocal laser scanning microscopy (CLSM) [21], and Raman spectroscopy [54,55]. They are incapable of providing numerical data but allow for the imaging of three-dimensional samples, such as skin, and information on the spatial distribution of the CAC in the different layers of the skin.

## 4. Membranes Mimicking Human Skin

Due to the ethical, health, and application problems associated with using human skin to study the permeation of CACs, alternative methods have been developed using different skin models. Various barriers have been sought to mimic human skin. Animal skin turned out to be an option because of its low cost and availability. From an anatomical point of view, the most similar to humans are pig, dog, or monkey skin. Often, analyses are carried out on rat, mouse, or rabbit skins that are structurally identical. However, several studies have shown that they are more permeable than human skin due to lower tissue thickness [56].

**Figure 5:**
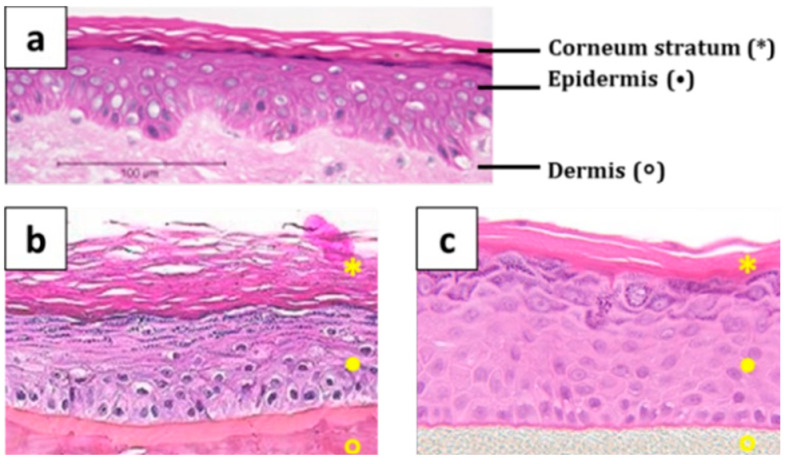
Comparison of a histological cross-section of (**a**) in vivo human skin, (**b**) EpiSkin, and (**c**) EpiDerm [57].

When biological skin is not readily available, synthetic membranes are employed for research. The great advantage of using these membranes is the reproducibility of the tests. In addition, they enable a much faster determination of the penetration properties and screening of a broader range of compounds. Recent developments in biotechnology and bioengineering have led to the implementation of advanced synthetic skin models, viz., 3D cell cultures (Figure 5). 

These models are based on cell cultures of human keratinocytes and show high structural similarity to natural tissues. Commercially available types of reconstructed human skin include EpiSkin, SkinEthic, EpiDerm, and Graftskin. However, these models are not commonly used due to the higher permeability of the simulated tissue than at in vivo conditions [58]. A summary of different skins and membranes used in the permeation tests is given in the Supplementary Materials; Table S1.

The best results on assessing the transdermal transport of CACs have been obtained for pig skin preparations, showing proximity to the data obtained for human skin. On the other hand, synthetic membranes display significantly poorer barrier properties than animal tissues. The models based on keratinocyte cell cultures also proved to be unsuitable for estimating the rate of transepidermal transport. The results were not comparable to human skin measurements.

In response to the lack of a suitable membrane capable of faithfully imitating human skin, EMD Millipore developed a synthetic skin model, the Strat-M^®^ membrane (see Supplementary Materials, Figure S1, for its structure) [59]. Strat-M^®^ makes it possible to predict the diffusion processes in human skin for various compounds and formulations, including CACs [60]. The most commonly used synthetic membrane models do not consider the biological composition of the stratum corneum, whereas the Strat-M^®^ membrane features a combination of lipids in a specific ratio similar to that found in the human stratum corneum. Moreover, this membrane is designed to preserve similar structural and chemical characteristics found in human skin (see below). However, it ignores any biological behavior due to the lack of viable cells [59,61].

The permeation tests using various biologically active compounds reveal comparable penetration behaviors for Strat-M^®^ and different skin models (Table S2). In addition, Kovács et al. [50] compared the permeation of diclofenac sodium from gels and creams across the Strat-M^®^ membrane, Skin-PAMPA, and heat-separated human epidermis. Similar permeation profiles recorded with the Strat-M and epidermis membranes allowed the authors to conclude that the former membrane is a better model than Skin-PAMPA. Strat-M^®^ also showed a closer similarity to human skin compared to pig and rat skin. Studies using microscopic techniques and Brunauer–Emmett–Teller analysis confirmed structural similarity between the Strat-M membrane and human skin in terms of thickness, pore size, and surface morphology [62].

Of other promising models for the human epidermis, Cerasome^®^9005 liposome suspensions should be mentioned due to their composition, which is similar to that of the stratum corneum. Cerasome^®^9005 comprises cholesterol, ceramides, fatty acids, and lecithin [63], and it has been used in diffusion cells to investigate the bioavailability of ionizing cosmetic raw materials in the presence of lipophilic counter ions and, importantly, copper complexes with low-molecular-weight peptides.

## 5. GHK–Cu and Evaluation of Its Skin Permeation

**Figure 6:**
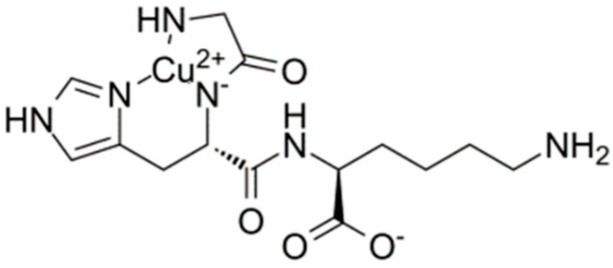
Structure of GHK–Cu [65].

Peptides are popular cosmetic ingredients as they immediately improve the appearance of the skin by moisturizing and conditioning its surface layer and exhibit additionally various biological effects. GHK (glycyl-L-histidyl-L-lysine) is a small, naturally occurring tripeptide with a high affinity toward the copper cation (Figure 6) [64]. The GHK tripeptide was first isolated in 1973 from human plasma by Loren Pickart and has been thereafter the subject of continuous research.

The GHK–Cu complex plays a crucial role in counteracting the signs of skin aging. Studies have shown that the concentration of GHK in the plasma of a 20-year-old is around 200 µg L^−1^, but its levels tend to decrease with age, reducing the skin’s ability to regenerate [66]. Its copper complex offers various health benefits, including promoting wound healing and skin regeneration, which inspired the interest among cosmetologists for many years. Owing to the diverse and well-documented biological effects on skin aging, GHK–Cu has earned the nickname “elixir of youth”. It was proven that GHK–Cu promotes tissue regeneration, stimulating collagen and decorin production. Moreover, it can increase skin density, smooth wrinkles, and even skin tone. GHK–Cu was also found to promote angiogenesis (the capillary formation), nerve growth, and DNA repair, and to pose antioxidant and anti-inflammatory effects [67].

**Figure 7:**
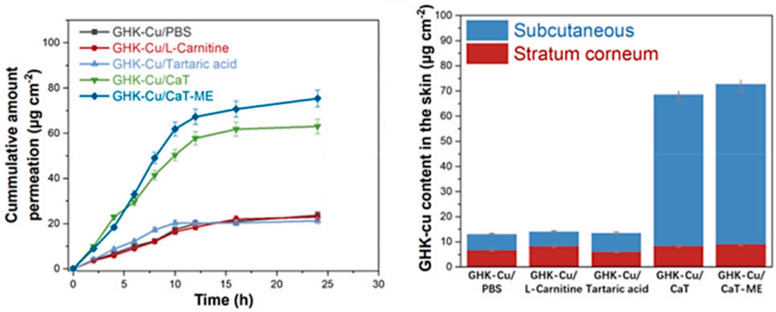
The influence of the type of GHK–Cu-mediated system on the skin retention and permeation of copper [71].

Although the GHK–Cu complex seems a “trendy” cosmetic component, its skin permeation has not been thoroughly characterized. So far, most tests have been performed using Franz or Flynn diffusion cells. Mazurowska and Mojski [68] studied the migration rate of GHK–Cu through the membrane imitating the stratum corneum to evaluate the ligand’s effect on copper permeation. The determined permeability coefficients confirmed that complexation boosts the permeation rate of copper ions. In a study by Hostynek et al., three different layers of skin from human cadavers (isolated stratum corneum, isolated epidermis, and dermatomed skin) were employed to portray the penetration of GHK–Cu as a potential anti-inflammatory agent [69]. The amount of copper passing through the individual layers was measured, and the retention was in the range of 0.6–2.8%. Liu et al. [70] developed a carrier based on an ionic liquid (IL) composed of betaine and tartaric acid, in which they encapsulated GHK–Cu and investigated its permeation through mice skin and its retention in its various layers. The presented results confirm that the IL enhances the ability of GHK–Cu to penetrate the skin and holds promise to improve its efficacy for applications like skin regeneration or antiaging treatments. In a follow-up report of the same group [71], a thermodynamically stable IL-based microemulsion (CaT-ME) was designed to better the transport of GHK–Cu across the skin barrier. The data shown in Figure 7 demonstrate that the parent IL (i.e., CaT) and, particularly, the microemulsion enable significantly better penetration of GHK–Cu than other systems such as L-carnitine or tartaric acid.

Table S3 gives a summary of skin permeation studies of GHK–Cu with detailed experimental conditions.

Returning to the question in the title, even though liposomes are commonly used as carriers to encapsulate and transport various active compounds due to their ability to improve skin penetration (see the following section), research on the permeation of GHK–Cu in this advanced form is still scarce. According to a few available contributions [12,13], the GHK–Cu is subject to encapsulation in liposomes, and its encapsulation efficiency can be determined using common analytical techniques like spectrophotometry or high-performance liquid chromatography (HPLC). However, these methods allow the encapsulated complex to be quantified only indirectly. Recently, an element-specific approach, based on inductively coupled plasma tandem mass spectrometry coupled with capillary electrophoresis (CE-ICP-MS/MS), was proposed to study the GHK–Cu–liposome systems without the need to separate the non-encapsulated compound from the liposomal suspension [17]. In this way, the encapsulation efficiency can be determined directly by measuring specific ^63^Cu^+^ signals from the GHK–Cu complex in liposomes and free GHK–Cu. Furthermore, CE-ICP-MS/MS makes it possible to simulate the physiological environment during the analysis. Nonetheless, the skin permeation of such systems remains a significant research gap. A critical success factor for resolving this challenge is the structural potential of liposomes to boost the efficacy of GHK–Cu, which could propel new possibilities for its application in cosmetology and regenerative medicine.

## 6. Skin Permeation of Active Ingredients Encapsulated in Liposomes

The growing interest in liposomes as carriers of CACs and cosmetics based on liposomal technology is linked to the need to advance methodologies for characterizing the liposomal CAC systems and studying their interactions with human skin. A key question is whether using such carriers affects the transport efficiency of the compound encapsulated inside them. Many compounds (vitamins, antioxidants, organic acids) have been encapsulated in liposomes, and their transportation through membranes has been studied to confirm their effectiveness (see Table 1; an extended version of this table, detailing pertinent experimental conditions, is shown in Table S4). Commonly used in these studies are FDSs. This standard approach is simple and inexpensive, and using skin-mimicking membranes and appropriate reagents (see Table S4) allows the physiological conditions to be imitated quite well. In this context, a solution with pH 7.4 is usually applied in the acceptor cell. On the other hand, the selection of static diffusion cells is not entirely justified, as the flow cells could be a better choice to account for the absorption of the compound in the blood circulatory system.

**Figure 8:**
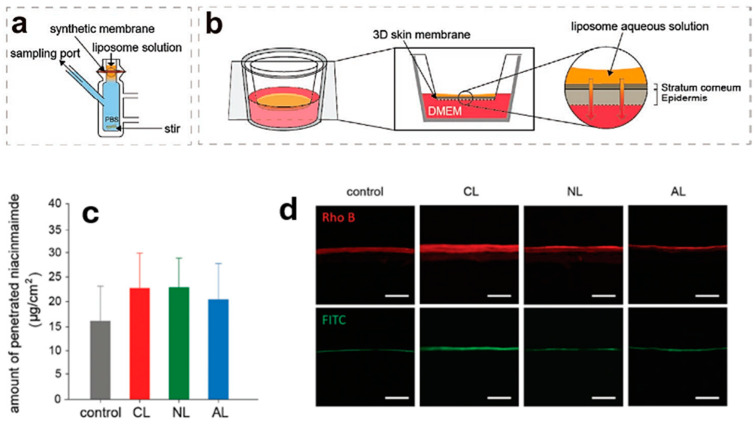
The scheme of (**a**) FDS and (**b**) 3D skin model experiments. Traces (**c**) and (**d**) show, respectively, the results of FDS experiments (24 h) and CLSM images of a cross-section of 3D skin treated with labeled liposomes. Legend: CLSM = confocal laser scanning microscopy; CL = cationic liposomes; NL = neutral liposomes; AL = anionic liposomes [74].

Although the literature reports collected in Table 1 are relatively recent, in most of them, merely conventional approaches rested on diffusion cells and human or animal skin find application. Only in a few contributions have new methodologies been attempted. Lee et al. [74] replaced natural skins with a synthetic Strat-M membrane to elucidate the effect of charged liposomes on the transdermal delivery of niacinamide. Also used in their study to visualize liposome permeation fluorescently was a 3D skin model, EpiDerm, featuring human dermal keratinocytes (Figure 8). Importantly, separate tests using the FDS and the membrane showed no significant differences. In contrast, the fluorescence intensities obtained with EpiDerm and labeled liposomes suggest increased permeability of niacinamide incorporated in cationic liposomes. The difference may be due to the fact that EpiDerm is a model of the human epidermis, whereas the synthetic membrane mimics all layers of the skin. Oh et al. also diverged from the adopted practice when employing the KeraSkin^TM^ model in their experiments along with human skin [73]. KeraSkin^TM^ is an epidermal model with a barrier function comparable to human skin due to its constitution of multilayered and fully differentiated human keratinocytes. Examinations on model and human skin confirmed the increased penetration of retinol delivered in deformable liposomes and revealed similar retinol permeation profiles.

Engesland and coworkers [52] performed acyclovir permeation tests from liposomal dispersions using the EpiSkin model and the phospholipid vesicle-based permeation assay (PVPA). In contrast to EpiSkin, the PVPA allows for tracking the differences in permeability that result from the form in which the drug is present. Palac et al. [80] used the PVPA barriers developed in Engesland’s study to find out how the physicochemical properties of liposomes affect the permeation of encapsulated sodium diclofenac. After modifying the barrier by reducing the thickness of the upper layer and changing the composition of the lipid vesicles, they observed a liposome-dependent transport and increased drug permeation (compared to the aqueous solution). Notably, the modified barrier was formed by vesicles prepared from components naturally present in the stratum corneum. It should be pointed out, however, that while PVPA is a simple, reproducible method alternative to diffusion cell testing, it provides no insight into the mechanism of liposome action, as the content of the vesicles in the acceptor compartment is unknown [80].

In addition to skin penetration, the retention of active ingredients in the individual layers of the skin can also be determined. This requires separating the individual layers and subsequent extraction of an active ingredient. The stratum corneum can be separated using adhesive tapes [10] and active ingredients extracted using a mixture of water and methanol acidified to pH 3.0 [7], methanol and hexane [9], ethanol [10], or SDS and NaCl solutions [81]. In each of the mentioned studies, encapsulating the compound in liposomes increased its accumulation in the skin. Skin retention is measured by the same analytical techniques as used for skin permeation assessment, e.g., HPLC or spectrophotometry. The former method improves the selectivity of measurements but organic solvents in common use are not friendly with the structure of liposomes. Also, neither technique is qualified for studying the mechanism of liposome action. Future research should therefore focus on finding a way to analyze the lipid vesicles quantitatively.

## 7. Conclusions

In this review, we raised a concern about the modern antiaging compound GHK–Cu with regard to improving its skin permeation. From the above discussion, it is evident that encapsulation in liposomes appears to be a cutting-edge approach for resolving this challenge that works well in the case of many other physiologically active compounds. The transport of various liposome-encapsulated entities through the skin has been scrutinized and appropriate methodologies have been developed to quantify skin permeation. Such a welcome situation affords grounds to hope that the enhanced uptake of GHK–Cu encapsulated in liposomes would become accessible before long. To realize this expectation, researchers should not only rely on existing procedures for measuring skin permeation but also create a reliable method to selectively determine GHK–Cu in the presence of liposomes and the encapsulated complex. In our opinion, high-resolution ICP-MS hyphenated with CE-(or other mild separation techniques) may fill the gap. However, its potential has not yet been sufficiently exploited in permeation studies and awaits in-depth examination. Another aspect of further research is understanding the mechanism of action of GHK–Cu encapsulated in liposomes, which would allow their properties to be fine-tuned to perform specific functions. Using synthetic membranes imitating human skin or live-skin equivalents is also essential as these models eliminate the need for human or animal tests and hence improve the method’s screening potential. When the effect of a liposomal carrier on the efficiency of GHK–Cu permeation through the epidermis and penetration into deeper skin layers is experimentally confirmed, this will be an upright starting point for fostering the liposomal GHK–Cu systems as cosmetic products.

## Figures and Tables

**Table 1:** Studies on skin permeation induced by liposomal encapsulation.

Active Compound	Skin Membrane	Measuring System	Main Results	Ref.
Vitamin C	Human skin	Jacketed FDSs/HPLC	Increased permeation (phosphatidylcholine liposomes)	[6]
	Pig ear skin	FDSs/HPLC	Increased vitamin stability, effectiveness, and skin permeation (negatively charged liposomes)	[7]
	Mouse skin	FDSs/UV-vis	Increased permeation (pectin-coated liposomes performed better than nanoliposomes)	[8]
Vitamin E	Pig skin	FDSs/UV-vis	Penetration increased in the order: macro emulsion < liposomes < liposomes dispersed in macro emulsion	[72]
Vitamin D3	Rat skin	FDSs/HPLC	Skin retention was 1.65 times higher for liposomal vitamin (than for vitamin in solution)	[9]
Retinol	Human skin and KeraSkin™	FDSs/spectrofluorimetry	Significantly increased permeation using Tween 20-based deformable liposomes (compared to conventional liposomes)	[73]
Niacinamide	Strat-M^®^	FDSs/HPLC	No significant differences in skin permeation depending on the type of liposomes	[74]
Coenzyme Q10	Rat dorsal skin	FDSs/HPLC	Higher skin deposition of encapsulated coenzyme	[10]
Resveratrol	Mouse dorsal skin	FDSs/HPLC	Coating the liposomes with chitosan increased their stability and improved drug permeation	[75]
Caffeine	Human skin	FDSs/HPLC	A two-fold increased permeation through the skin (compared to caffeine solution)	[11]
Naringin	Pig skin	FDSs/HPLC	No significant effect of liposomes on skin permeation	[76]
Taxifolin	HuSKIN	FDSs/UV-vis	Deformable liposomes containing polyglyceryl-2 caprate showed effective carrier functions	[77]
Hyaluronic acid	Human skin	Vertical FDS/HPLC	Enhanced drug release after incorporating Tween^®^80 or Transcutol^®^P into liposomal formulation	[78]
Salicylic acid	Porcine skin	FDSs/UV-vis	Two-fold increases in skin permeation and retention (compared to gel formulation)	[79]

## Data Availability

Not applicable.

## Supplementary Materials

The following supporting information can be downloaded at: https://www.mdpi.com/article/10.3390/molecules30010136/s1, Figure S1: Structure of the Strat-M^®^ membrane; Table S1: Selected permeation tests; Table S2: Different skins comparable to Strat-M^®^ in terms of drug penetration ability; Table S3: Skin permeation studies of GHK–Cu; Table S4: Studies on skin permeation induced by liposomal encapsulation [82,83,84,85,86,87,88,89,90,91,92,93,94,95,96,97,98,99,100].
