# Supplementary material for: Are We Ready to Measure Skin Permeation of Modern Antiaging GHK–Cu Tripeptide Encapsulated in Liposomes?
Source: Molecules. 2025 Jan 1;30(1):136. doi: 10.3390/molecules30010136 (PMC11721469; doi:10.3390/molecules30010136)
Supplement: Supplementary file 1 [file molecules-30-00136-s001.zip › molecules-3328545-supplementary.pdf]

Supporting Materials for

Are We Ready to Measure the Skin Permeation of Modern Antiaging GHK–Cu Tripeptide Encapsulated in Liposomes?

Karolina Ogórek [1], Kinga Nowak [1], Emilia Wadych [1], Lena Ruzik [1], Andrei R. Timerbaev [2,*], and Magdalena Matczuk [1,*]

[1] Chair of Analytical Chemistry, Warsaw University of Technology, Noakowskiego St. 3, 00-664 Warsaw, Poland; K.N. (kinga.nowak3.stud@pw.edu.pl), E.W. (emilia.wadych.stud@pw.edu.pl), L.R. (lena.ruzik@pw.edu.pl)
[2] Institute of Inorganic Chemistry, University of Vienna; Währinger Str. 38, 1090 Vienna;
* Correspondence: magdalena.matczuk@pw.edu.pl (M.M.), andrei.timerbaev@univie.ac.at (A.R.T.)

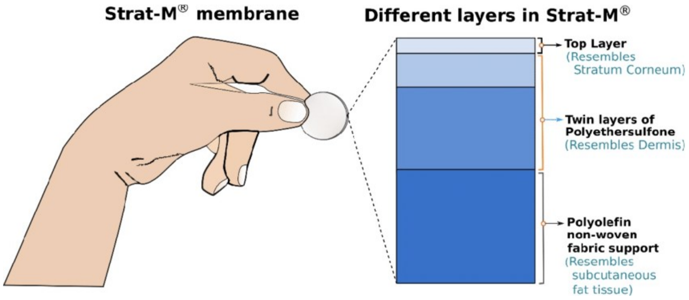

Figure S1. Structure of the Strat-M® membrane [1].

Table S1. Selected permeation tests.

Permeation Entity	Membrane	Ref.
Salicylic acid from ointment	PDMS membrane	[2]
Hydrocortisone, testosterone and progesterone	Cellulose acetate membrane	[3]
Drugs in ionic liquids	Milipore HAWP, Cuprophan	[4]
Nitroglycerine	P-HEMA/styrene membrane	[5]
Salicylic acid, hydrocortisone, clotrimazole, and terbinafine	Graftskin, SkinEthic	[6]
Caffeine and tocopherol acetate from w/o emulsion, o/w, liposomal dispersion, and hydrogel	EpiSkin, EpiDerm, SkinEthic	[7]
4-Methylbenzylidene camphor microspheres	EpiSkin	[8]
Nanoemulsion containing methotrexate	Pig ear skin	[9]

Urea	Human skin	[10]
1-Phenoxy-2-propanol in shampoo and cream	Rat skin	[11]
Substance P-based hydrogel	KeraSkin®-FT	[12]
Carfentanil	EpiDerm	[13]

Table S2. Different skins comparable to Strat-M® in terms of drug penetration ability.

**Active Compound**	**Skin Model**	**Ref.**
Rivastigmine	Porcine skin	[14]
Diclofenac sodium	Human epidermis	[15]
Nicotine	Human epidermis	[16]
Lidocaine	Human skin	[17]
Caffeine	Porcine skin	[18]
Collagen peptide	Mouse skin	[19]

Table S3. Skin permeation studies of GHK–Cu.

The aim of the study	Skin Membrane	Method of studying skin permeation	Method of quantitative determination of active compound	Ref.
Transport of GHK–Cu from aqueous solution through the model membrane	Lipophilic membrane modelling stratum corneum lipids – Cerasome®9005 (Lipoid GmbH, Germany) placed between two polyester foil discs	Side-by-side Flynn diffusion cell<br>▪ Conditions: 25 °C, 1000 rpm;<br>▪ Donor cell: 27 mL of phosphate buffer, pH 7.4 containing copper complex;<br>▪ Acceptor cell: 15 mL of phosphate buffer solution, pH 7.4;<br>▪ Diffusion area: 1.77 cm^2.	UV-vis (copper determination)<br>▪ Sample preparation: 1 mL of the solution from acceptor cell + 1 mL of 0.1% cuprizon solution + 2 mL of ammonium buffer, pH 10.0 + 6 mL of water;<br>▪ Detection wavelength: 600 nm.	[20]
Transport of GHK–Cu from O/W emulsion through the model membrane	Lipophilic membrane modelling stratum corneum lipids – Cerasome®9005 (Lipoid GmbH, Germany) placed between two polyester foil discs	Franz diffusion cell<br>▪ Conditions: 25 °C, 1000 rpm;<br>▪ Donor cell: 1 g of an O/W emulsion containing copper complexes with peptides;<br>▪ Acceptor cell: 15 mL of phosphate buffer solution, pH 7.4;<br>▪ Diffusion area: 1.77 cm^2.	UV-vis (copper determination)<br>▪ Sample preparation: 1 mL of the solution from acceptor cell + 1 mL of 0.1% cuprizon solution + 2 mL ammonium buffer, pH 10.0 + 6 mL of water;<br>▪ Detection wavelength: 600 nm.<br><br>RPLC-UV-vis (tripeptide determination)<br>▪ Analytical column: Hypersil BDS C18 (4.0 x 125 mm);<br>▪ Sample volume: 20 μL;<br>▪ Mobile phase: 0,15% trifluoroacetic acid solution;<br>▪ The flow rate of mobile phase: 0.7 mL min^{-1};<br>▪ Detection wavelength: 200 nm.	[21]

Evaluation of skin retention and penetration of GHK–Cu	Human cadaver skin; different layers	<u>Glass low-volume flow cells</u><br>▪ Conditions: 37 °C;<br>▪ Donor cell: 1 mL of 0.68% GHK–Cu in aqueous solution;<br>▪ Acceptor cell: distilled water containing 1 mL L^{-1} of gentamicin sulfate solution (100 mg mL^{-1});<br>▪ Acceptor medium flow rate: 2 mL h^{-1}<br>▪ Diffusion area: 0.8 cm^2.	<u>ICP-MS</u><br>▪ Sample dilution: 1:10, v:v; in 2% HNO_3;<br>▪ Collision gas: helium;<br>▪ Internal standard: ^{89}Y;<br>▪ Monitored isotopes: ^{63}Cu, ^{65}Cu.	[22]
Evaluation of transdermal permeation of GHK–Cu encapsulated in an ionic-liquid-based carrier	Mouse skin	<u>Franz diffusion cell</u><br>▪ Conditions: 32 ± 2 °C, 320 rpm;<br>▪ Donor cell: GHK–Cu encapsulated in an ionic-liquid-based carrier;<br>▪ Acceptor cell: PBS buffer, pH 6.8;<br>▪ Diffusion area: 1.0 cm^2.	<u>HPLC-DAD</u><br>no data	[23]
Evaluation of GHK–Cu permeation depending on used carrier (PBS, L-carnitine, tartaric acid, CaT, and CaT-ME)	Porcine skin	<u>Franz diffusion cell</u><br>▪ Conditions: 32 °C, 300 rpm;<br>▪ Donor cell: ionic liquid (IL)-based microemulsion (IL-M) with GHK-Cu;<br>▪ Acceptor cell: 0.01 mol L^{-1} PBS buffer;<br>▪ Diffusion area: 1.0 $cm^{2.}$.	<u>HPLC-UV</u><br>▪ Analytical column: C18 column (ZORBAX Eclipse);<br>▪ Sample volume: 5 μL;<br>▪ Mobile phase: water + methanol + 0.1% aqueous trifluoroacetic acid;<br>▪ The flow rate of mobile phase: 1.0 mL min^{-1};<br>▪ Detection wavelength: 220 nm.	[24]

Table S4. Studies on skin permeation induced by liposomal encapsulation.

Active Compound	Skin Membrane	Method of studying skin permeation	Method of quantitative determination of active compound	Ref.
**Vitamins and their derivatives**				
	Human skin	Franz diffusion jacketed cells<br>▪ Conditions: 36 ± 1 °C, 500 rpm;<br>▪ Donor cell: liposomal formulations containing ascorbic acid or free ascorbic acid solution;<br>▪ Acceptor cell: 5 mL of phosphate-buffered saline, pH 7.4;<br>▪ Diffusion area: 0.9 cm^2.	HPLC-UV<br>▪ Analytical column: Spherisorb ODS1 with precolumn C18;<br>▪ Sample volume: 100 μL;<br>▪ Mobile phase: potassium hydrogen phosphate buffer, pH 2.6;<br>▪ The flow rate of mobile phase: 1.0 mL min^{-1};<br>▪ Detection wavelength: 254 nm.	[25]
Vitamin C	Pig ear skin	Franz diffusion cells<br>▪ Conditions: 32 °C, 300 rpm;<br>▪ Donor cell: 0.2 mL of liposome formulation containing ascorbic acid or free ascorbic acid solution;<br>▪ Acceptor cell: 6.6 mL of 0.1 mol L^{-1} succinate buffer adjusted with phosphoric acid to pH 3.0, containing 0.02% sodium thiosulfate;<br>▪ Diffusion area: 1.86 cm^2.	HPLC-DAD<br>▪ Analytical column: C18 250 × 4.5 mm, 5μm Zorbax XDB column, and C18 12.5 × 4.6 mm Zorbax XDB pre-column;<br>▪ Sample volume: 20 μL;<br>▪ Mobile phase: 10 mmol L^{-1} tetrabutylammonium hydrogen sulfate solution;<br>▪ The flow rate of mobile phase: 1.0 mL/min;<br>▪ Detection wavelength: 245 nm.	[26]
	Mouse skin	Franz diffusion cells<br>▪ Conditions: 37 ± 1 °C, 500 rpm;<br>▪ Donor cell: 1 mL of different formulation;<br>▪ Acceptor cell: 4.0 mL of physiological solution;<br>▪ Diffusion area: 1.77 cm^2.	UV-vis<br>▪ Sample preparation: 0.2 mL of receptor solution was mixed with 5 mL methanol and shaken for 10 min; 1 mL of previously processed mixture + 0.3 mL of 0.25 mol L^{-1} EDTA, 0.5 mL of 0.5 mol L^{-1} acetic acid + 1.25 mL of fast blue B salt (2 g L^{-1}) + 6.95 mL of water;<br>▪ Detection wavelength: 430 nm.	[27]
Vitamin E	Pig skin	Franz diffusion cells<br>▪ Conditions: 25 ± 0.5 °C;<br>▪ Donor cell: 1000 μL of macro emulsion containing tocopherol in liposomes;<br>▪ Acceptor cell: phosphate buffered saline (PBS);	UV-vis<br>▪ Sample preparation: 2000 μL of receptor solution + 2000 μL of methanol;<br>▪ Detection wavelength: 291.4 nm	[28]

Vitamin D3	Rat skin	Franz diffusion cells<br>▪ Conditions: 37 °C, 400 rpm;<br>▪ Donor cell: 1 mL of liposomes containing vitamin D3 or vitamin D3 solution;<br>▪ Acceptor cell: saline solution containing 30% (v/v) ethanol;<br>▪ Diffusion area: 2.54 cm^2.	HPLC-UV<br>▪ Analytical column: C18 reversed-phase column (4.6 × 250 mm, 5 µm);<br>▪ Mobile phase: methanol;<br>▪ The flow rate of mobile phase: 1 mL min^{-1};<br>▪ Detection wavelength: 204 nm.	[29]
Retinol	Human skin and KeraSkin™	Franz diffusion cells<br>▪ Conditions: 37 ± 0.5 °C, 500 rpm;<br>▪ Donor cell: 300 µL of retinol-containing liposomes or free retinol;<br>▪ Acceptor cell: 12 mL of phosphate-buffered solution, pH 7.0;<br>▪ Diffusion area: 1.77 cm^2.	Spectrofluorimetry<br>▪ Sample preparation: 100 µL of sample + 2.5 mL 1% Triton X-100 in phosphate buffered saline;<br>▪ Excitation wavelength: 395 nm;<br>▪ Emission wavelength: 485 nm.	[30]
Niacinamide	Strat-M®	Franz diffusion cells<br>▪ Conditions: 37 ± 0.5 °C, 300 rpm;<br>▪ Donor cell: 0.2 mL of niacinamide-containing liposomes;<br>▪ Acceptor cell: 7 mL of phosphate-buffered saline (PBS);<br>▪ Diffusion area: 1.766 cm^2.	HPLC-UV<br>▪ Analytical column: reversed phase column Gemini 5 µm C18, 4.6 × 250 mm);<br>▪ Mobile phase: 10 mmol L^{-1} KH_2PO_4 in water:acetonitrile (93:7);<br>▪ The flow rate of mobile phase: 1 mL min^{-1};<br>▪ Detection wavelength: 263 nm.	[31]
**Antioxidants**				
Coenzyme Q10	Rat dorsal skin	Franz diffusion cells<br>▪ Conditions: 37 °C, 100 rpm;<br>▪ Donor cell: 200 µl of the vesicular formulations or solution of coenzyme Q10; dissolved in 2% Tween 80;<br>▪ Acceptor cell: 7.5 mL of phosphate buffer, pH 7.4;<br>▪ Diffusion area: 1.76 cm^2.	HPLC-UV<br>▪ Analytical column: C-18 column;<br>▪ Mobile phase: 1-propanol:methanol 60:40, v/v containing 89.5 mmol L^{-1} perchloric acid and 57 mmol L^{-1} sodium hydroxide;<br>▪ The flow rate of mobile phase: 1 mL min^{-1};<br>▪ Detection wavelength: 275 nm.	[32]

Resveratrol	Mouse dorsal skin	Franz diffusion cells<br>▪ Conditions: 37 °C;<br>▪ Donor cell: 0.2 mL of liposomes containing resveratrol, or coated-liposomes containing resveratrol;<br>▪ Acceptor cell: 5 mL of HCO60:ethanol:phosphate buffered saline (PBS), 2:20:78, w/w/w.	HPLC-UV<br>▪ Analytical column: Shim-pack VP-ODS C18 (250 mm x 4.6 mm)	[33]
Caffeine	Abdominal full-thickness human skin	Franz diffusion cells<br>▪ Conditions: 35 °C;<br>▪ Donor cell: 160 µL of the liposome formulations, or the control solutions;<br>▪ Acceptor cell: 3.5 mL of PBS, pH 7.4;<br>▪ Diffusion area: 1.33 cm^2.	HPLC-UV<br>▪ Analytical column: Phenomenex Luna C18 5µm (150 × 4.6 mm);<br>▪ Mobile phase: 95.5% water, 2% acetonitrile, 2% tetrahydrofuran and 0.5% acetic acid;<br>▪ The flow rate of mobile phase: 1 mL min^{-1};<br>▪ Detection wavelength: 273 nm.	[34]
Naringin	Pig skin	Franz diffusion cells<br>▪ Conditions: 32 °C;<br>▪ Donor cell: 800 µL of ultradeformable liposomes containing naringin;<br>▪ Acceptor cell: buffer phosphate solution, pH 7.4;<br>▪ Diffusion area: 0.784 cm^2.	HPLC-UV<br>▪ Analytical column: Teknokroma® Brisa “LC2” C18, 5.0 µm (150 cm x 4.6 mm);<br>▪ Mobile phase: a mixture of 50:50 (v/v) of methanol and ultrapure water, pH 4.0;<br>▪ The flow rate of mobile phase: 1 mL min^{-1};<br>▪ Detection wavelength: 280 nm.	[35]
Taxifolin	HuSKIN	Franz diffusion cells<br>▪ Conditions: 37 °C, 150 rpm;<br>▪ Donor cell: taxifolin– and taxifolin tetraoctanoate–loaded liposomes;<br>▪ Acceptor cell: HCO-60:ethanol:PBS ¼,), 2:20:78, w/w/w;<br>▪ Diffusion area: 0.784 cm^2.	UV-vis<br>▪ Detection wavelength: 290 nm (taxifolin)<br>▪ Detection wavelength: 280 nm (taxifolin tetraoctanoate)	[36]
**Organic acids**				
Hyaluronic acid	Human skin	Vertical Franz-type diffusion cells<br>▪ Conditions: 32 ± 0.5 °C;<br>▪ Donor cell: 300 µL of different formulations containing hyaluronic acid in liposomes;	HPLC-UV<br>▪ Analytical column: C18 reverse-phase column (Kromasil 100 C18 5 µm, 25 × 0.46 cm^2);	[37]

		▪ Acceptor cell: MilliQ water or Hepes buffer, pH 7.4;<br>▪ Diffusion area: 0.6 cm^2.	▪ Mobile phase: a mixture of water and acetonitrile (98:2, v/v);<br>▪ The flow rate of mobile phase: 0.5 mL min^{-1};<br>▪ Detection wavelength: 195 nm.	
Salicylic acid	Porcine skin	<u>Franz diffusion cells</u><br>▪ Conditions: 37 ± 0.5 °C;<br>▪ Donor cell: salicylic acid-loaded liposomal gel or the salicylic acid plain gel;<br>▪ Acceptor cell: 15 mL of phosphate buffer, pH 5.5;<br>▪ Diffusion area: 2.53 cm^2.	<u>UV-vis</u><br>▪ Detection wavelength: 296 nm	[38]
	Porcine skin	<u>Franz diffusion cells</u><br>▪ Conditions: 37 °C;<br>▪ Donor cell: 2.0 mL of dispersion containing salicylic acid in liposomes or dispersion containing 2% w/w plain salicylic acid;<br>▪ Acceptor cell: 33 mL of phosphate buffer, pH 5.0;<br>▪ Diffusion area: 2.269 cm^2.	<u>UV-vis</u><br>▪ Detection wavelength: 295.2 nm	[39]

References

1. Ponmozhi, J.; Dhinakaran, S.; Varga-Medveczky, Z.; Fónagy, K.; Bors, L.; Ivan, K.; Erdő, F. Development of Skin-On-A-Chip Platforms for Different Utilizations: Factors to Be Considered. *Micromachines* **2021**, *12*, 294.
2. Nakano, M.; Patel, N.K. Release, uptake and permeation behaviour of salicylic acid in ointment bases. *Journal of Pharmaceutical Sciences* **1970**, *59*(7), 985–988.
3. Barry, B.W.; El Eini, D. Solubilization of hydrocortisone, dexamethasone, testosterone and progesterone by long-chain polyoxyethylene surfactants. *Journal of Pharmacy and Pharmacology* **1976**, *28*(3), 210–218.
4. Lee, S.J.; Kurihara-Bergstrom, T.; Kim, S.W. Ion-paired drug diffusion through polymer membranes. *International Journal of Pharmaceutics* **1987**, *39*(1–2), 59–73.
5. Minghetti, P.; Casiraghi, A.; Cilurzo, F.; Montanari, L.; Marazzi, M.; Falcone, L. Donati, V. Comparison of different membranes with cultures of keratinocytes from man for percutaneous absorption of nitroglycerine. *Journal of Pharmacy and Pharmacology* **1999**, *51*, 673–678.
6. Schmook, F.P.; Meingassner, J.G.; Billich, A. Comparison of human skin or epidermis models with human and animal skin in in vitro percutaneous absorption. *International Journal of Pharmaceutics* **2001**, *215*(1–2), 51–56.
7. Netzlaff, F.; Lehr, C.M.; Wertz, P.W.; Schaefer, U.F. The human epidermis models EpiSkin, SkinEthic and EpiDerm: An evaluation of morphology and their suitability for testing phototoxicity, irritancy corrosivity, and substance transport. *European Journal of Pharmaceutics and Biopharmaceutics* **2005**, *60*(2), 167–178.
8. Monti, D.; Chetoni, P.; Burgalassi, S.; Tampucci, S.; Centini, M.; Anselmi, C. *4-Methylbenzylidene camphor microspheres: reconstituted epidermis (Skinethic®) permeation and distribution. International Journal of Cosmetic Science* ***2015***, *37*(3), 298–305.
9. Rajitha, P.; Shammika, P.; Aiswarya, S.; Gopikrishnan, A.; Jayakumar, R.; Sabitha, M. Chaulmoogra oil based methotrexate loaded topical nanoemulsion for the treatment of psoriasis. *Journal of Drug Delivery and Science Technology* **2019**, *49*, 463–476.
10. Intarakumhaeng, R.; Alsheddi, L.; Wanasathop, A.; Shi, Z.; Li, S.K. Skin permeation of urea under finite dose condition. *Journal of Pharmaceutical Sciences* **2019**, *129*, 173–180.
11. Lee, J.D.; Kim J.Y.; Jang, H.J.; Lee, BM.; Kim, KB. Percutaneous permeability of 1-phenoxy-propanol, a preservative in cosmetics. *Regulatory Toxicology and Pharmacology* **2019**, *103*, 56–62.
12. Kim, D.J.; Chang, S.S.; Lee, J. Anti-Aging Potential of Substance P-Based Hydrogel for Human Skin Longevity. *International Journal of Molecular Sciences* **2019**, *20*, 4453.
13. Lenta, E.M.; Maistrosb, K. J.; Oyler, J.M. In vitro dermal absorption of carfentanil. *Toxicology in Vitro* **2020**, *62*, 104696.
14. Simon, A.; Amaro, M.I.; Healy, A.M.; Cabral, L.M.; Pereira de Sousa, V. Comparative evaluation of rivastigmine permeation from a transdermal system in the Franz cell using synthetic membranes and pig ear skin with in vivo-in vitro correlation. *International Journal of Pharmaceutics* **2016**, *512*, 234-241.
15. Haq, A.; Dorrani, M.; Goodyear, B.; Joshi, V.; Michniak-Kohn, B. Membrane Properties for Permeability Testing: Skin versus Synthetic Membranes. *International Journal of Pharmaceutics* **2018**, *539*, 58-64.
16. Haq, A.; Goodyear, B.; Ameen, D.; Joshi, V.; Michniak-Kohn, B. Strat-M synthetic membrane: Permeability comparison to human cadaver skin. *International Journal of Pharmaceutics* **2018**, *547*(1), 432-437.
17. Zsikó, S.; Cutcher, K.; Kovács, A.; Budai-Szucs, M.; Gácsi, A.; Baki, G.; Csányi E.; Berkó, S. Nanostructured Lipid Carrier Gel for the Dermal Application of Lidocaine: Comparison of Skin Penetration Testing Methods. *Pharmaceutics* **2019**, *11*, 310.
18. Pulsoni, I.; Lubda, M.; Aiello, M.; Fedi, A.; Marzagalli, M.; Hagen, J.; Scaglione, S. Comparison between Franz Diffusion Cell and a novel Micro-physiological system for in vitro penetration assay using different skin models. *SLAS Technology* **2022**, *27*, 161-171.
19. Yan, Z.; Dong, P.; Jiang, B.; Zhang, XQ.; Wang, HY. An evaluation of collagen peptide for transdermal delivery using Strat-M® membrane and excised mouse skin. *Acad. J. Pham. Pharmacol*. **2024**, *11*(1), 001-008.
20. Mazurowska, L.; Mojski, M. ESI-MS study of the mechanism of glycyl-l-histidyl-l-lysine-Cu(II) complex transport through model membrane of stratum corneum. *Talanta* **2007**, *72*(2), 650–654.
21. Mazurowska, L.; Mojski, M. Biological activities of selected peptides: Skin penetration ability of copper complexes with peptides. *J. Cosmet. Sci.* **2008**, *59*, 59-69.
22. Hostynek, J.J.; Dreher, F.; Maibach, H.I. Human skin retention and penetration of a copper tripeptide in vitro as function of skin layer towards anti-inflammatory therapy. *Inflamm. Res*. **2010**, *59*(11), 983–988.
23. Liu, T.; Hu, L.; Lu, B.; Bo, Y.; Liao, Y.; Zhan, J.; Pei, Y.; Sun, H.; Wang, Z.; Guo, C.; Zhang J. A novel delivery vehicle for copper peptides. *New Journal of Chemistry*. **2023**, *47*, 75-83.
24. Liu, T.; Liu, Y.; Zhao, X. Thermodynamically stable ionic liquid microemulsions pioneer pathways for topical delivery and peptide application. *Bioactive Materials*. **2024**, *32*, 502-513.

25. Serrano, G.; Almudéver, P.; Serrano, JM.; Milara, J.; Torrens, A.; Expósito I.; Cortijo, J. Phosphatidylcholine liposomes as carriers to improve topical ascorbic acid treatment of skin disorders. *Clinical, Cosmetic and Investigational Dermatology* **2015**, *8*, 591–599.
26. Maione-Silva, L.; de Castro, E.G.; Nascimento, T.L.; Cintra, E.R.; Moreira, L.C.; Cintra, B.A.S.; Valadares, M.C.; Lima E.M. Ascorbic acid encapsulated into negatively charged liposomes exhibits increased skin permeation, retention and enhances collagen synthesis by fibroblasts. *Scientific Reports* **2019**, *9*, 522.
27. Zhou, W.; Liu, W.; Zou, L.; Liu, W.; Liu, C.; Liang, R.; Chen, J. Storage stability and skin permeation of vitamin C liposomes improved by pectin coating. *Colloids and Surfaces B: Biointerfaces* **2014**, *117*, 330–337.
28. Karunaratne, N.; Dassanayake, A.; Pamunuwa, G.; Karunaratne, V. Improved skin permeability of dl-α-tocopherol in topical macroemulsions. *International Journal of Pharmacy and Pharmaceutical Sciences* **2014**, *6*(6), 53–57
29. Bi, Y.; Xia, H.; Li, L.; Lee, R.J.; Xie, J.; Liu, Z.; Qiu, Z.; Teng, L. Liposomal Vitamin D3 as an Antiaging Agent for the Skin. *Pharmaceutics* **2019**, *11*, 311.
30. Oh, YK.; Kim, M.Y.; Shin, JY.; Kim, T.W.; Yun, MO.; Yang, S.J.; Choi, S.S.; Jung, WW.; Kim, J.A.; Choi, H.G. Skin permeation of retinol in Tween 20-based deformable liposomes: in-vitro evaluation in human skin and keratinocyte models. *Journal of Pharmacy and Pharmacology* **2006**, *58*, 161–166.
31. Lee, M.S.; Lee, J.W.; Kim, S.J.; Pham-Nguyen, OV.; Park, J.; Park, J.H.; Jung, Y.M.; Lee, J.B.; Yoo, H.S. Comparison Study of the Effects of Cationic Liposomes on Delivery across 3D Skin Tissue and Whitening Effects in Pigmented 3D Skin. *Macromolecular Bioscience* **2021**, *21*, 2000413.
32. El-Zaafarany, G. M.; Abdel-Aziz, R. T. A.; Montaser, M. H. A.; Nasr, M. Coenzyme Q10 phospholipidic vesicular formulations for treatment of androgenic alopecia: ex vivo permeation and clinical appraisal. *Expert Opinion on Drug Delivery* **2021**, *18*(10), 1513–1522.
33. Park, S.N.; Jo, N.R.; Jeon, S.H. Chitosan-coated liposomes for enhanced skin permeation of resveratrol. *Journal of Industrial and Engineering Chemistry* **2014**, *20*, 1481–1485.
34. Abd, E.; Gomes, J.; Sales, C.C.; Yousef, S.; Forouz, F.; Telaprolu, K.C.; Roberts, M.S.; Grice, J.E.; Lopes, P.S.; Leite-Silva, V.R.; Andréo-Filho, N. Deformable liposomes as enhancer of caffeine penetration through human skin in a Franz diffusion cell test. *International Journal of Cosmetic Science* **2021**, *43*, 1–10.
35. Pleguezuelos-Villa, M.; Mir-Palomo, S.; Díez-Sales, O.; M.A. Ofelia Vila Buso, Ruiz Sauri, A.; Nácher, A. A novel ultradeformable liposomes of Naringin for anti-inflammatory therapy. *Colloids and Surfaces B: Biointerfaces* **2018**, *162*, 265–270.
36. Kim, A.R.; An, H.J.; Jang, E.S.; Lee, J.D.; Park, S.N. Preparation, Physical Characterization, and In Vitro Skin Permeation of Deformable Liposomes Loaded with Taxifolin and Taxifolin Tetraoctanoate. *European Journal of Lipid Science and Technology* **2019**, *121*, 1800501.
37. Vázquez-González, M.L.; Calpena, A.C.; Domènech, O.; Montero, M.T.; Borrell, J.H. Enhanced topical delivery of hyaluronic acid encapsulated in liposomes: A surface-dependent phenomenon. *Colloids and Surfaces B: Biointerfaces* **2015**, *134*, 31–39.
38. Chandwani, S.; Saini, T. R.; Soni, R.; Paswan, S. K.; Soni, P. K. Box-Behnken design optimization of salicylic acid loaded liposomal gel formulation for treatment of foot corn. *International Journal of Applied Pharmaceutics* **2023**, *15*(3), 220–233
39. Bhalerao, S. S.; Raje Harshal, A. Preparation, Optimization, Characterization, and Stability Studies of Salicylic Acid Liposomes. *Drug Development and Industrial Pharmacy* **2003**, *29*(4), 451–467.
